# Association between ambient cold exposure and mortality risk in Shandong Province, China: Modification effect of particulate matter size

**DOI:** 10.3389/fpubh.2022.1093588

**Published:** 2023-01-05

**Authors:** Zhonghui Zhao, Jie Chu, Xiaohui Xu, Yanwen Cao, Tamara Schikowski, Mengjie Geng, Gongbo Chen, Guannan Bai, Kejia Hu, Jingjing Xia, Wei Ma, Qiyong Liu, Zilong Lu, Xiaolei Guo, Qi Zhao

**Affiliations:** ^1^Department of Epidemiology, School of Public Health, Shandong University, Jinan, China; ^2^Shandong University Climate Change and Health Center, Jinan, China; ^3^Shandong Center for Disease Control and Prevention, Jinan, China; ^4^Academy of Preventive Medicine, Shandong University, Jinan, China; ^5^Department of Epidemiology, Leibniz Institute for Environmental Medicine (IUF)-Leibniz Research Institute for Environmental Medicine, Düsseldorf, Germany; ^6^Chinese Center for Disease Control and Prevention, Beijing, China; ^7^Department of Occupational and Environmental Health, School of Public Health, Sun Yat-sen University, Guangzhou, Guangdong, China; ^8^Department of Child Health Care, The Children's Hospital, National Clinical Research Center for Child Health, Zhejiang University School of Medicine, Hangzhou, China; ^9^Department of Big Data in Health Science, School of Public Health, Zhejiang University, Hangzhou, China; ^10^School of Life Sciences, Greater Bay Area Institute of Precision Medicine (Guangzhou), Fudan University, Guangzhou, China

**Keywords:** particulate matter, ambient cold, modification effect, mortality, air pollution

## Abstract

**Introduction:**

Numerous studies have reported the modification of particulate matters (PMs) on the association between cold temperature and health. However, it remains uncertain whether the modification effect may vary by size of PMs, especially in Shandong Province, China where the disease burdens associated with cold temperature and PMs are both substantial. This study aimed to examine various interactive effects of cold exposure and ambient PMs with diameters ≤1/2.5 μm (PM1 and PM2.5) on premature deaths in Shandong Province, China.

**Methods:**

In the 2013-2018 cold seasons, data on daily mortality, PM1 and PM2.5, and weather conditions were collected from the 1822 sub-districts of Shandong Province. A time-stratified case-crossover study design was performed to quantify the cumulative association between ambient cold and mortality over lag 0-12 days, with a linear interactive term between temperature and PM1 and PM2.5 additionally added into the model.

**Results:**

The mortality risk increased with temperature decline, with the cumulative OR of extreme cold (−16.9°C, the 1st percentile of temperature range) being 1.83 (95% CI: 1.66, 2.02), compared with the minimum mortality temperature. The cold-related mortality risk was 2.20 (95%CI: 1.83, 2.64) and 2.24 (95%CI: 1.78, 2.81) on high PM1 and PM2.5 days, which dropped to 1.60 (95%CI: 1.39, 1.84) and 1.60 (95%CI: 1.37, 1.88) on low PM1 and PM2.5 days. PM1 showed greater modification effect for per unit concentration increase than PM2.5. For example, for each 10?g/m3 increase in PM1 and PM2.5, the mortality risk associated with extreme cold temperature increased by 7.6% (95% CI: 1.3%, 14.2%) and 2.6% (95% CI: −0.7%, 5.9%), respectively.

**Discussion:**

The increment of smaller PMs' modification effect varied by population subgroups, which was particularly strong in the elderly aged over 75 years and individuals with middle school education and below. Specific health promotion strategies should be developed towards the greater modification effect of smaller PMs on cold effect.

## 1. Introduction

Extreme temperature events are increasingly frequent in the context of climate change, becoming a major health challenge now and in the next decades (1). Numerous studies have reported that exposure to cold or high temperatures are both associated with adverse health outcomes. Despite the global warming trend, most temperature-related excess deaths are still attributable to exposure to cold temperatures (2, 3). For example, it is estimated that 9.43% of global premature deaths are associated with non-optimal temperature, with 8.52% explainable by cold exposure (4).

Particulate matter (PM) is another major environmental hazard, triggering a broad range of health outcomes such as cardiovascular disease, respiratory dysfunction and premature deaths (5–7). In 2019, exposure to PMs caused 6.4 million premature deaths, accounting for 18.4% of the global total mortality and therefore ranking the fifth top health risk factor (8). Moreover, PMs with smaller size may be more vulnerable than coarse PMs (9–11). For example, in Anhui Province, China between 2016 and 2018, the risk of hospitalization on childhood pneumonia increased by 10.28, 1.21, and 1.10% for each 10 μg/m^3^ increase in PMs with a diameter of ≤1.0, 2.5, and 10 μm (i.e., PM_1_, PM_2.5_, and PM_10_), respectively (9).

In recent years, increasing public concerns have focused on the combined effect of ambient cold and PMs. A study in Guangzhou, China has reported that the cold-related mortality risk may increase by 40.7 and 46.7% on days with PM_10_ concentrations at the 25^th^ and 75^th^ percentiles of concentration range during study period, respectively (12). Another European study has found that the cold-related risk of cardiovascular mortality may increase by 16.2 and 2.0% on days with high and low particle number concentrations (PNCs), respectively (13). However, it remains largely unclear how the modification effect of PMs on cold-related health risk may vary by the size of PMs. Clarifying this research issue can help us better understand the mixed disease burden of the two environmental factors, and thus develop more targeted health promotion strategies.

China has one of the greatest disease burdens from PMs and ambient suboptimal temperature (14). The lancet countdown report and the national environmental surveillance have further indicated that the PMs' concentration and extreme temperature events are more substantial in Shandong province than many other Chinese areas, considering its the second largest population size, unique location, and rapid population aging trend (15). Shandong has abundant industrial activities and a coal-based energy structure. In China, although the concentration of PM_2.5_ has declined in recent years, the PM pollution remain heavy in Shandong Province in comparison to most other regions (16). This leads Shandong Province to be an ideal area to evaluate the combined health risk of ambient cold and various sizes of PMs.

Using data from 1,822 sub-districts in Shandong Province, China, this study is designed to explore how PMs with various sizes (i.e. PM_1_ and PM_2.5_) may modify the strength of association between ambient cold temperature and mortality.

## 2. Materials and methods

### 2.1. Study area

Shandong Province is a coastal province in eastern China that locates between 34.382° N - 38.400° N and 114.792° E - 122.705° E, and covers an area of 155,800 km^2^. It has a monsoon climate of medium latitudes with four distinct seasons throughout the year. In 2021, the resident population in Shandong Province was 101.5 million, ranking it the second largest population size in China.

### 2.2. Data collection

We collected daily deaths in 1,822 sub-districts of Shandong province from 2013 to 2018 from Shandong Center for Disease Control and Prevention. Variables included each individual's date of death, age, gender, education level, cause of death (coded using the international classification of diseases code, ICD-10) and home address.

The daily concentrations of PM_1_ and PM_2.5_ were modeled at a spatial resolution of 0.01° × 0.01°, with the methods on modeling well described by our previous studies and the data quality validated (17, 18). Briefly, we collected daily data on PMs from monitoring sites of the China National Environmental Monitoring Center. A random forest model based on machine learning algorithm was combined with big data to generate data sets, including ground measurements, satellite remote sensing products, and atmospheric reanalysis. The daily meteorological data during the same period were collected from the China Meteorological Data Network (http://data.cma.cn/) at a spatial resolution of 0.01° × 0.01°, including daily average temperature, relative humidity and wind velocity. Meteorological data and pollution data were assigned to the sub-district where the individual lived.

### 2.3. Statistical analysis

Following previous studies (19–21), the cold season was defined as the four consecutive coldest months of the year in Shandong province. A time-stratified case-crossover study design was applied: Conditional logistic regression with the non-distributed lag model was used to fit the relationship between ambient temperature and risk of mortality in the cold season. Specifically, each case works as a stratum, and its controls were defined as the days of the same week of the same year and month (22). This self-matched case-control design can adjust for temporal variation and the effect of time-dependent variables such as age, sex and lifestyle (23). The bidirectional selection of controls can reduce long-term temporal trend, seasonality, and confounding effect caused by the “day-of-the-week effect.”

Our initial analysis indicated that the adverse effect of ambient temperature lasted for up to 12 days, which then were used in the formal analysis and justified in the sensitivity analysis below. The equation was as follows:

*Logit* (*P*) = α__*stratum*_(i)_ + *cb* (Temp_i_) + ns(RH_i_, df=3)+ PM_i_ + *cb* (Temp_i_)^*^PM_i_.

Where *P* is the possibility of deaths. Each death case (i) with its controls works as a strata, and α_1_ to α_*s*_ are stratum constants (assuming that the number of deaths is s). *cb* (Temp_i_) is the crossbasis function obtained by DLNM for the death case i to fit the nonlinear and lagged effect of temperature, with a natural cubic spline with three degrees of freedom (dfs) for the temperature dimension and a natural cubic spline with four dfs for the lag dimension. ns(RH_i_, df = 3) represents a natural spline function with 3 df for the moving average value of relative humidity (RH) over lag 0–12 days. PM_i_ is the linear function of moving average value of PM_1_ or PM_2.5_ over lag 0–12 days. The *cb* (Temp_i_)^*^PM_i_ is the interactive term between the crossbasis function of temperature and the linear function of PM_1_ or PM_2.5_.

Two model sets were used to examine the modification effects of PM_1_ and PM_2.5_ on temperature-mortality risk, respectively. For each model set, the interactive term was entered into the model by centering PM_1_ or PM_2.5_ at the low, median and high levels, respectively. Following previous studies (13, 24, 25), the low, median and high levels of PM_1_ and PM_2.5_ were defined using relative thresholds, i.e., the concentrations at the 10^th^, 50^th^ and 90^th^ percentile of the PM_1_ and PM_2.5_ concentration range during the study period. The extreme cold effect was defined as the cumulative OR (lag 0–12 days) with 95% confidence interval (CI) of mortality corresponding to the 1^st^ percentile of the temperature range in the cold season, compared with the minimum mortality temperature (MMT).

To straightforwardly compare the modification effects of PM_1_ and PM_2.5_, the percentage changes in risk of mortality associated with extreme cold temperature for each 10 μg/m^3^ increase in PM_1_ and PM_2.5_ were calculated from the coefficient of the interactive term. Stratified analyses were performed by gender, age-groups (< 75 yr or ≥75 yr), educational level (middle school and below, or above middle school), and by cause of death [cardiorespiratory diseases (I00-J99) and others (A00-B99, C00-D48, D50-H95 and K00-Z99)].

### 2.4. Sensitivity analyses

We conducted a series of sensitivity analyses to verify the robustness of the model and parameters, including by changing the maximum lag of temperature from 10 to 18 days, and the positions and number of knots in the crossbasis function, the dfs in the natural cubic spline function of relative humidity from three to four, and by additionally adding the moving average value of ozone over lag 0–12 days into the model.

All analyses were conducted using R software (version 4.1.2). The “dlnm” package and “mvmeta” package were used to fit distributed lag non-linear model and multivariate meta-analysis, respectively.

## 3. Results

### 3.1. Descriptive statistics

**Figure 1:**
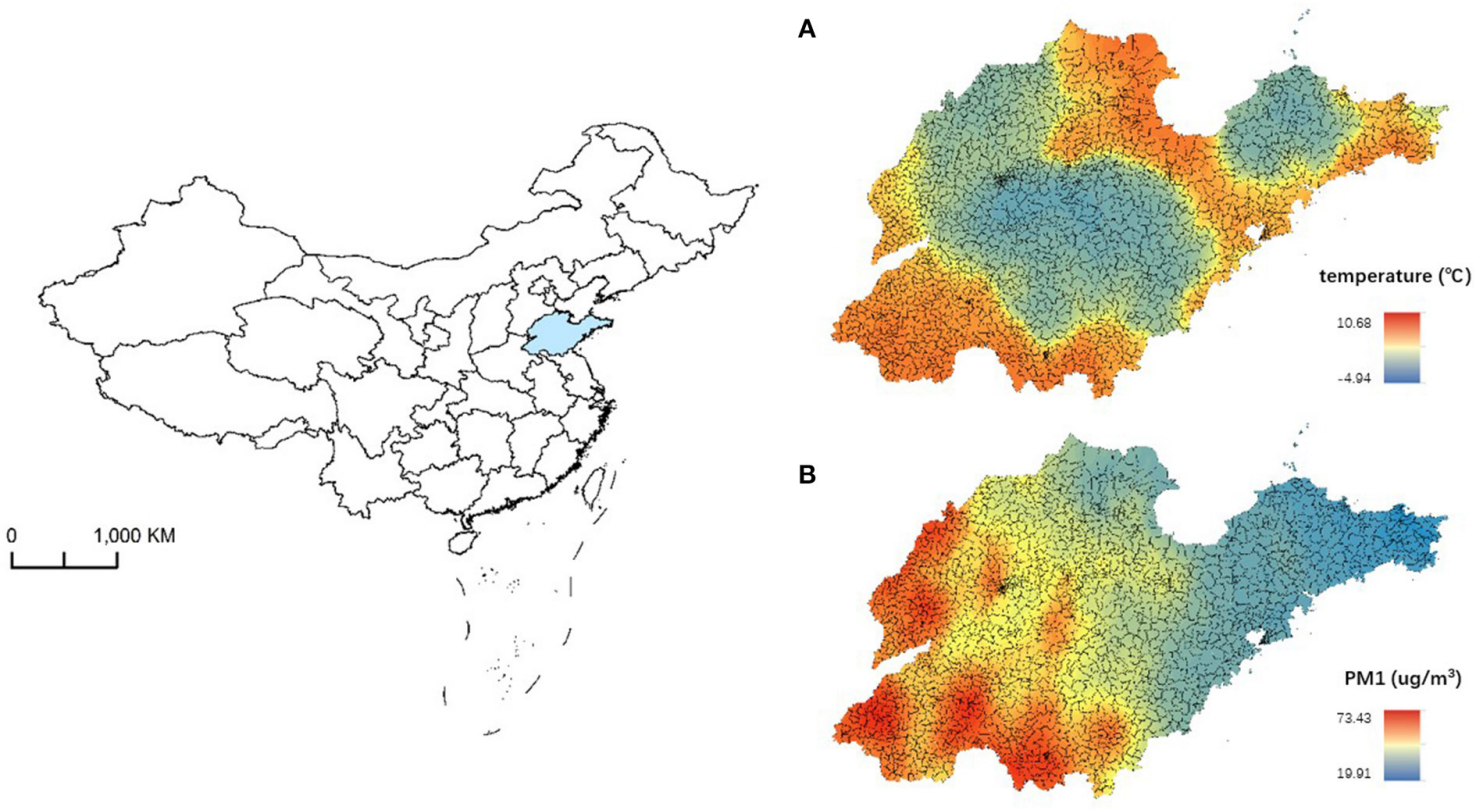
The distribution of temperature **(A)** and PM_1_ concentration **(B)** in cold season in Shandong Province during 2013–2018.

From 2013 to 2018, 1,473,300 deaths were recorded in the cold season (November to February) in all sub-districts of Shandong Province (Table 1). The median values of ambient temperature, PM_1_ and PM_2.5_ (with 25^th^ and 75^th^ percentile range) in the cold season were 1.17 (−1.49, 4.37)°C, 51.78 (42.63, 63.78) μg/m^3^, 81.34 (56.33, 111.05) μg/m^3^, respectively (Figure 1 and Supplementary Figure S1).

**Table 1:** Demographic and environmental characteristics of mortality in cold season in Shandong Province from 2013 to 2018.

**Characteristic**		**Value (%)**
Demographic characteristics		
Gender	Male	824,601 (55.97)
	Female	648,699 (44.03)
Age	< 75	649,725 (44.10)
	≥75	823,575 (55.90)
Educational level	Middle school and below	1,320,387 (89.62)
	Above middle school	77,241 (5.24)
	Missing value	75,672 (5.14)
Cause of death	Cardiorespiratory diseases	944,106 (64.08)
	Other	526,194 (35.92)
Air pollutants percentiles		
PM_2.5_ (μg/m^3^)	50^th^ (25^th^, 75^th^)	81.34 (56.33, 111.05)
PM_1_ (μg/m^3^)	50^th^ (25^th^, 75^th^)	51.78 (42.63, 63.78)
Air temperature percentiles (°C)	50^th^ (25^th^, 75^th^)	1.17 (-1.49, 4.37)

### 3.2. Cold death effect under different pollution levels

**Figure 2:**
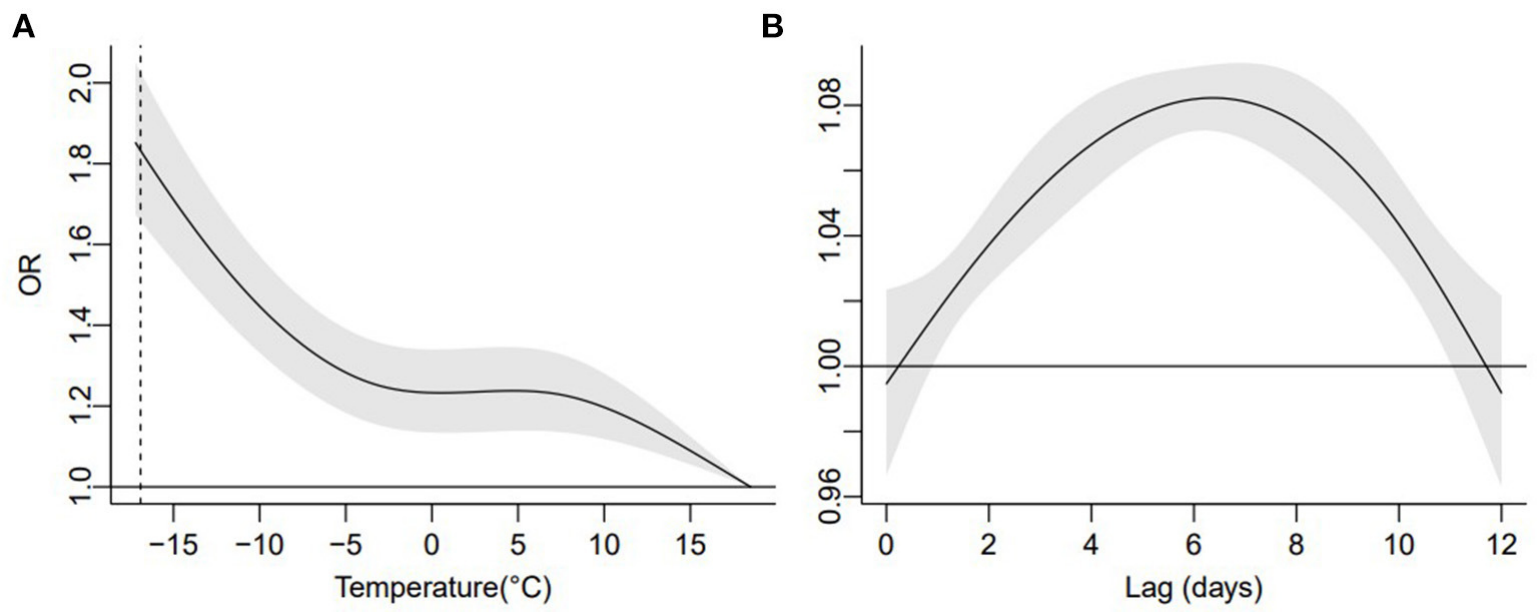
Overall cumulative relationship between ambient temperature and mortality **(A)** in the cold season, and the associated lag-response pattern **(B)**. The dotted line indicates extreme low temperature.

**Figure 3:**
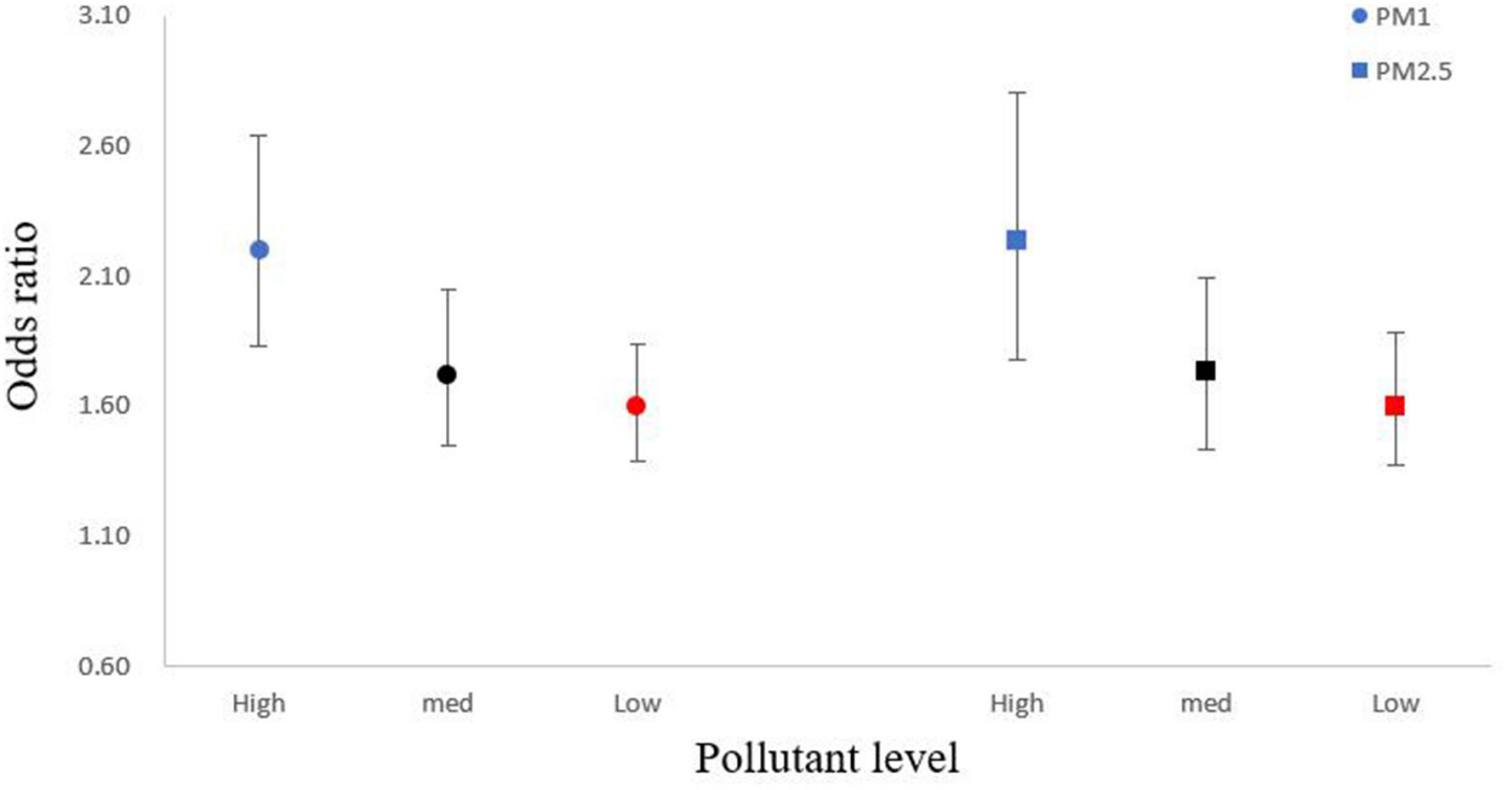
Modified overall cumulative air temperature-mortality associations at extreme low temperature by air pollution with 95% CIs. In the abscissa “pollution level,” the “high” represent for high air pollution level (concentration above 90^th^ value), the “med” represent for medium air pollution level (concentration for 50^th^ value), the “low” represent for low air pollution level (concentration below 10^th^ value).

The risk of all-cause mortality increased with temperature cooling, with the MMT located at 18.5°C (Figure 2). The effect of extreme cold effect, i.e., the cumulative OR of mortality at the extreme low temperature (−16.9°C), was 1.83 (95% CI: 1.66, 2.02) over lag 0–12 days, in comparison to the MMT. In general, the cold-related risk of mortality was stronger at high levels of PM_1_ and PM_2.5_ (Figure 3). For example, the cumulative extreme cold effect was 2.20 (95%CI: 1.83, 2.64) and 2.24 (95%CI: 1.78, 2.81) on days with high PM_1_ and PM_2.5_ concentrations in the cold season. The extreme cold effect dropped to 1.60 (95%CI: 1.39, 1.84) and 1.60 (95%CI: 1.37, 1.88) on days with low PM_1_ and PM_2.5_ concentrations. Lag pattern analysis for PM_1_ and PM_2.5_ had similar results (Supplementary Figure S2): the extreme cold effect combined with high PMs appeared on the first day of cold exposure and diminished until lag 11 day, which commonly appeared on lag 2 day and disappeared until lag 10 day for the combination of cold temperature, and low PM_1_ and PM_2.5_ levels.

### 3.3. The modification effect of PMs and stratified analysis

Results indicated that the modification effect was slightly higher for smaller PMs size, such that for every 10 μg/m^3^ in PM_1_ and PM_2.5_, the mortality risk associated with extreme cold temperature increased by 7.6% (95% CI: 1.3%, 14.2%) and 2.6% (95% CI: −0.7%, 5.9%), respectively. The modification effect of smaller PMs on cold temperature-mortality association varied across population subgroups (Table 2). For example, for every 10 μg/m^3^ in PM_2.5_ and PM_1_, the increment of mortality risk associated with extreme cold temperature increased from 3.4% (95% CI: −1.2%, 8.1%) to 9.9% (95% CI: 0.9%, 19.8%) for the elderly aged over 75 years, which remained insignificant for younger adults. The variation was more obvious for subgroups with different educational levels: for every 10 μg/m^3^ in PM_2.5_ and PM_1_, the increment of mortality risk associated with extreme cold temperature increased from 3.4% (95% CI: −0.3%, 7.4%) to 8.3% (95% CI: 1.4%, 15.7%) for individuals with middle school education or below, which remained insignificant with the effect size also nearly unchanged for individuals with middle school educational level or higher.

**Table 2:** Percentage change in cold-related mortality per 10 μg/m^3^ increase in PM_1_ and PM_2.5_ in Shandong Province from 2013 to 2018.

		**PM_1_**	**PM_2.5_**
Gender	Male	7.8 (−0.8, 17.0)	3.8 (−0.5, 8.2)
	Female	7.7 (−1.4, 17.6)	1.5 (−3.0, 6.2)
Age	≥75	9.9 (0.9, 19.8)	3.4 (−1.2, 8.1)
	< 75	5.8 (−2.6, 14.8)	1.5 (−3.0, 6.3)
Educational level	Above middle school	1.0 (0.3, 1.8)	0.3 (0.0, 0.6)
	Middle school and below	8.3 (1.4, 15.7)	3.4 (−0.3, 7.4)
Cause of death	Cardiorespiratory diseases	5.7 (−2.3, 14.3)	1.3 (−2.7, 5.6)
	Other	13.7 (3.6, 24.8)	5.6 (0.7, 10.8)
	Overall	7.6 (1.3, 14.2)	2.6 (−0.7, 5.9)

In this study, the extreme cold effect was defined as the cumulative OR with 95% (CI) of mortality corresponding to the 1^st^ percentile of the temperature range in the cold season. An interactive term between the crossbasis function of cold temperature and the linear function of PM_2.5_ or PM_1_ was added into the model. To straightforwardly compare the modification effects of PM_1_ and PM_2.5_, the percentage changes in risk of mortality associated with extreme cold temperature for each 10 μg/m^3^ increase in PM_1_ and PM_2.5_ were calculated from the coefficient of the interactive term. To make the definitions of cold effect and the modification effect of PMs clearer, we have added the aforementioned sentences into the footnote of this table.

### 3.4. Sensitivity analysis

Sensitivity analysis indicated the results were reliable by changing lags of temperature, and positions and number of knots in the crossbasis function, and by additionally adding ozone into the model (Supplementary Figures S3–S5).

## 4. Discussion

Our study in Shandong Province of China indicated a strong association between ambient cold temperature and risk of mortality. The strength of association was strongly intensified by high PMs concentrations, with smaller size of PMs showing greater modification effect. Subgroup analysis indicated that the stronger modification effect of smaller PMs on cold-mortality association varied across population subgroups, which was particularly obvious for the elderly aged 75 years and above, and for individuals educated middle school and below.

Previously, numerous studies have reported the modification of PMs on adverse effect of suboptimal temperature while most of them focused on heat exposure. For example, in France, every 10 μg/m^3^ increase in PM_10_ was associated with a 14.2% increase in mortality during heat waves (26). In comparison, less evidence is available on the combined impact of PMs and cold temperature. Our study observed a strong association between ambient temperature in the cold season and mortality risk in Shandong Province, with the strength of association greater on days with high PMs concentrations. Similar findings were reported in Europe, where cold temperature was observed to have a greater impact on total mortality and cardiovascular mortality on days with high PMs levels (13). Another Chinese study also showed that the death risk of PM_2.5_ at low temperature was higher than that at medium temperature (27). Cold temperatures can lead to vasoconstriction and the release of inflammatory markers in the blood, which may increase blood pressure and vascular resistance, and finally trigger cardiovascular events (28). In comparison to previous explorations, our analysis provides details of the changing cold effect by PMs levels over lag days. The extreme cold effect combined with high PMs appeared immediately rather than appeared on lag 2 day with low PMs levels, and diminished until lag 11 day. This may indicate that the PM level has a specific modification on the extreme cold effect. In the case of high particulate pollution, the extreme cold effect would appear directly after cold exposure. It is speculated that low temperature and PM exposures may share certain pathways toward adverse health outcomes, such as reducing the clearance rate of respiratory mucosa cilia (29). This may partly explain the immediate cold effect on days with high PM concentration. Further research is encouraged to clarify the underpinning mechanism.

Some studies have evaluated whether the health impact of PM may vary by its physical size. For example, a study has pointed out that the global risk of all-cause mortality may increase by 0.44 and 0.68% per 10 μg/m^3^ increase in PM_10_ and PM_2.5_, respectively (30). A study in Guangzhou, China has indicated that PM_1_ may be a more important risk factor for cardiovascular death (31). Another Chinese study in Zhejiang Province has also shown that PM_1_ have slightly higher impacts on all-cause, cardiovascular and respiratory mortality than PM_2.5_ (10). In addition, a mice experiment has observed that the toxic effect of PM_2.5_ on lung tissue cells increases with exposure to higher PM_2.5_ mass concentration, and PM_1_ may explain the major effect size of PM_2.5_ in damaging mouse cells (32). In comparison, the modification of PMs size on cold effect remains largely unclear. Our study found that smaller size of PMs might have greater modification effect on the association between ambient cold and mortality.

Without more clinical and molecular information, it is difficult to explain the underpinning mechanisms of the observed higher modification effect of PM_1_ than PM_2.5_. However, compared with PM_2.5_, PM_1_ has small size and large active surface area, allowing it to penetrate the deepest part of the respiratory system and then travel to other physiological organs *via* the circulatory system in an easier way (33). As a result, the health risk is expected to be greater under the same exposure concentration of PM_1_ than for PM_2.5_ (34). A study shows that cold temperature can impair the antiviral response that induced by interferon (35). Both PMs and cold temperature are reported to cause inflammation: PMs exposure induces mucus metaplasia and increases mucus production (36). Meanwhile, cold temperature can induce airway inflammation and excess mucus production through cold-inducible RNA-binding protein mediated increase in mRNA stability and protein translation (37). Another study has indicated that low ambient temperature and PM may increase the level of respiratory inflammation (38). The evidence aforementioned may explain the interactive effect between cold temperature and PM exposure, and the higher modification effect of smaller size of PM. However, more supportive findings are still necessary to clarify the mechanism.

Stratified analysis showed that the greater modification effect of PM_1_ than PM_2.5_ on cold-related mortality risk was more obvious for certain population subgroups such as the elderly aged over 75 years and individuals with middle school education and below. There is no substantial difference in the modification effect of PMs between males and females. Few studies have explored the differences between men and women in the modification effect of PMs. However, a study in Hong Kong found that although the cold-related mortality risk for both males and males was significantly higher, the gender difference in cold effect is the smallest (39). In addition, although the mortality risk of PM_2.5_ in females was slightly stronger than males, the differences were not statistically significant (40). Consistent with our study, previous research found that with the rise of particulate matter levels, the elderly are more vulnerable to low temperature than the young (12). In addition, we found that the modification effect of PMs on cold-related mortality risk was higher for other diseases than cardiorespiratory disease. The mechanism remains unclear without more details about the study population at the individual level. However, a considerable proportion of patients in “Other” group were due to tumors, whose physiological systems were extremely susceptible to cold temperature and air pollution because many studies showed that low temperature and PMs are risk factors for tumors (41–44).

We found that individuals of low educational level were more susceptible to low temperatures, and the risk of death increased with the rise of PMs concentration. Many studies have reached similar conclusion (45, 46). Compared with well-educated people, less educated people are more vulnerable to extreme temperature events (47). Socioeconomic factors such as educational attainment may modify the health effects of air pollution in several pathways (48). For example, it is speculated that individuals of low educational level are often at a lower socioeconomic status, which may lead to poor living standards and nutritional conditions.

Our study has several strengths. First, we used a time stratified case-crossover design to explore the various interactive effects of cold exposure and ambient PMs on premature deaths, which can control both seasonal effect and day-of-the-week effect. The self-control method has well balanced the impact of many individual factors on the outcome. Second, compared with other studies, we focused on exploring the effects of different size particles on the cold temperature-death relationship, which help better understand the combined effects of air pollution and extreme temperature events. Finally, most studies exploring the individual or combined effect of air pollutants and ambient temperature using data at the city or county level. In this study, the assignment of pollution and weather exposure at a sub-district level improved the accuracy of exposure assessment. There are several limitations to this study. We were unable to consider the impact of PM components on the cold-mortality association. In addition, the environmental exposure assessment still needs improvement in the future, such as at the individual level.

## 5. Conclusions

Our study finds that the risk of cold temperature on mortality increased on days with higher PMs concentrations, and the modification effect increased for smaller size of PMs. The findings suggest that more consideration should be given to the combined effect of smaller air pollution particles and ambient cold when developing healthcare strategies. Specific subgroups, such as individuals aged 75 years and above, and those with low educational level should be considered with public health priority.

## Data availability statement

The datasets presented in this article are not readily available because data is confidential. Requests to access the datasets should be directed to QZ, qi.zhao@sdu.edu.cn.

## Author contributions

ZZ: conceptualization, methodology, formal analysis, data curation, and writing–original draft. JC, XX, TS, MG, GC, GB, KH, and JX: writing–review and editing. YC: methodology and formal analysis. WM and QL: conceptualization, supervision, and writing–review and editing. ZL: data curation. XG: data curation and writing–review and editing. QZ: conceptualization, methodology, writing–review and editing, supervision, project administration, and funding acquisition. All authors contributed to the article and approved the submitted version.
