# Supplementary material for: Association between ambient cold exposure and mortality risk in Shandong Province, China: Modification effect of particulate matter size
Source: Front Public Health. 2023 Jan 5;10:1093588. doi: 10.3389/fpubh.2022.1093588 (PMC9850236; doi:10.3389/fpubh.2022.1093588)
Supplement: Supplementary file 1 [file Data_Sheet_1.docx]

Supplemental Information

**Association between ambient cold exposure and mortality risk in Shandong Province, China: Modification effect of particulate matter size**


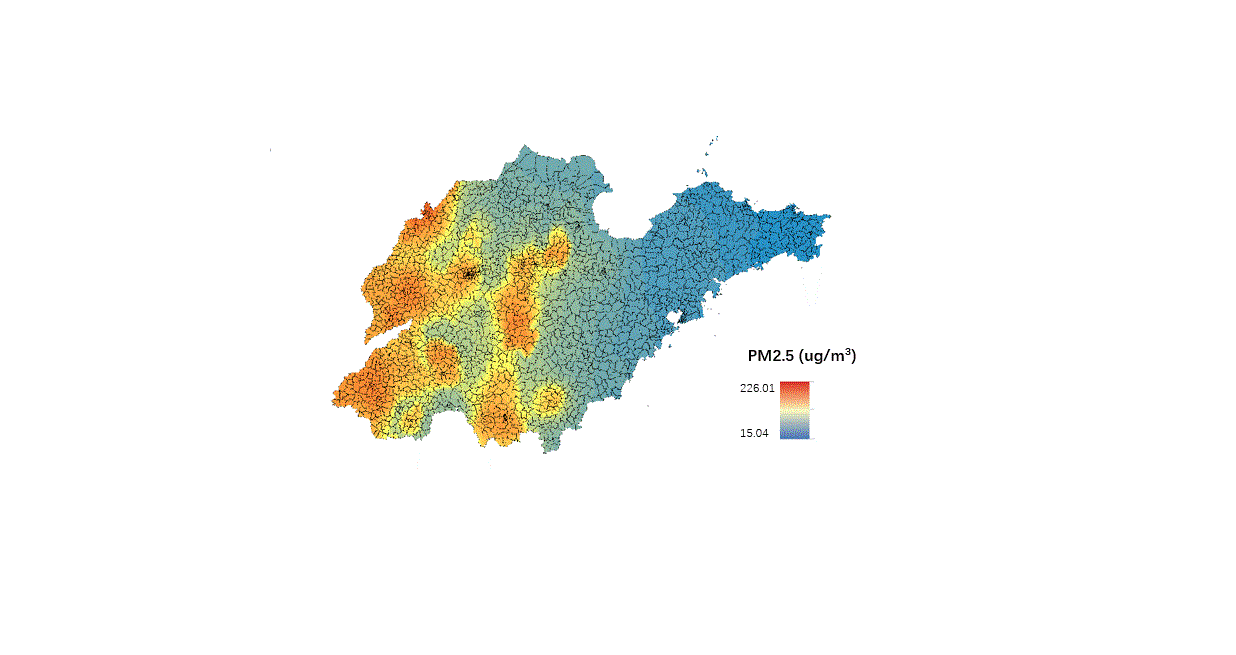


Fig. S1. The distribution of PM_2.5_ concentration in cold season in Shandong Province during 2013-2018.


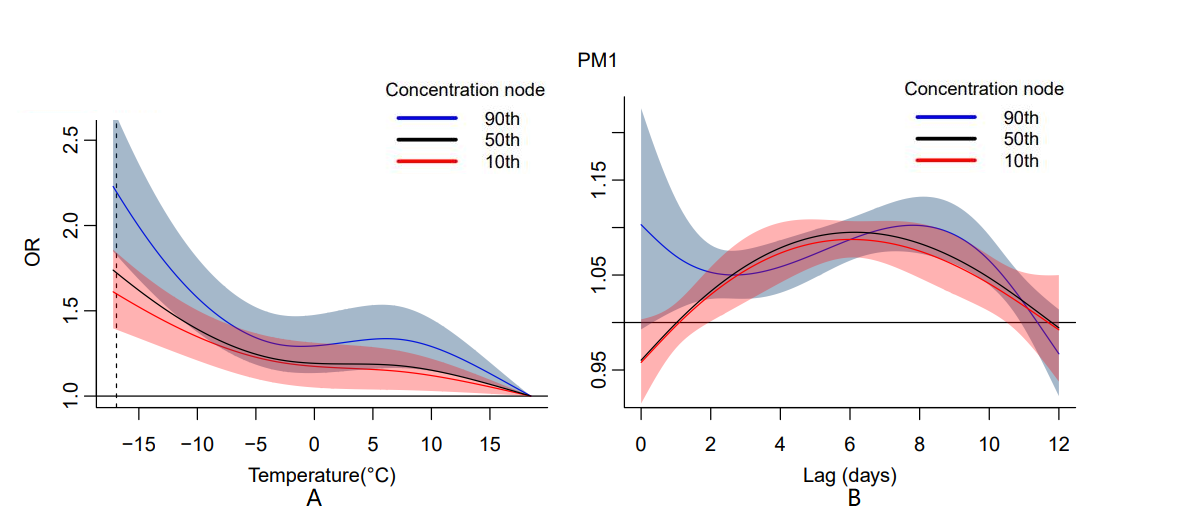

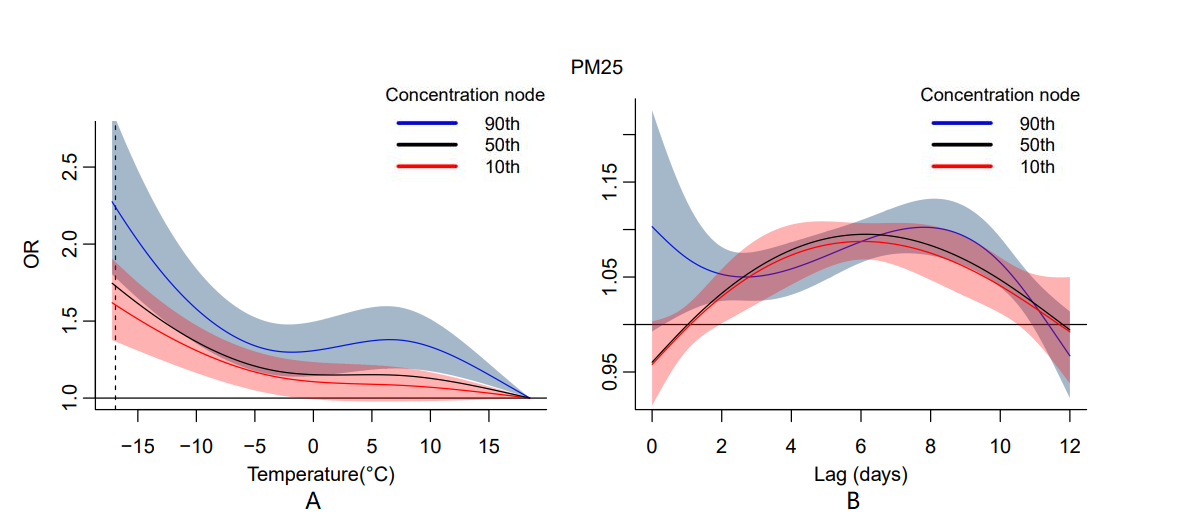


Fig. S2. Modified relationships between cold season temperature and death with 95% CIs by PM_1_ and PM_2.5_. Overall cumulative relationship between ambient temperature and mortality under different PMs levels (A) in the cold season, and the associated lag-response pattern(B). The dotted line indicates extreme low temperature. Blue curve represents the effect of PMs concentrations were at the 90^th^ percentile of range in the cold season, black curve represents the effect of PMs concentrations were at the 50^th^ percentile of range in the cold season and red curve represents the concentrations at the 10^th^ percentile of range.


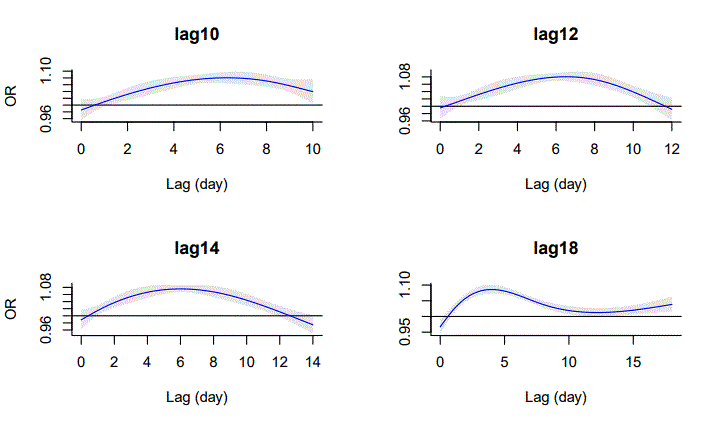
Fig. S3. Results of sensitivity analyses by changing maximum lags from 10 to 18 days.


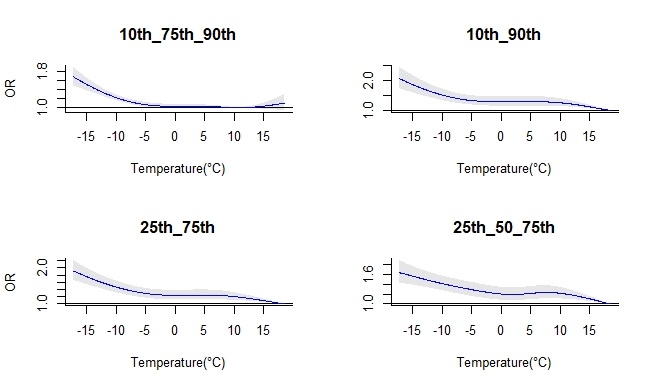


Fig. S4. Results of sensitivity analyses by changing positions and number of knots in the crossbasis function.


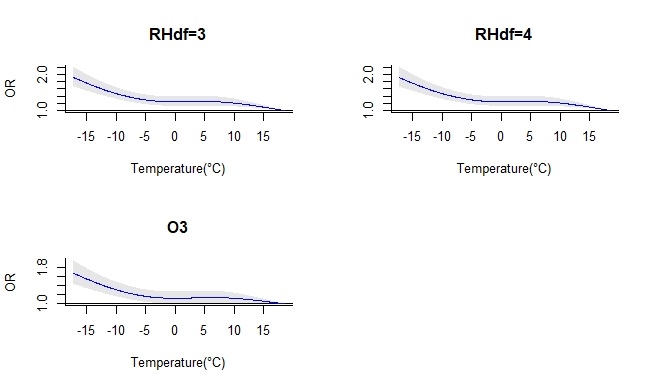


Fig. S5. Results of sensitivity analyses by changing degree of freedom (df) in the natural cubic spline function of relative humidity from three to four, and additionally adding the moving average value of ozone over lag 0-12 days into the model.
